# Plasma D-dimer levels are associated with disease progression in diabetic nephropathy: a two-center cohort study

**DOI:** 10.1080/0886022X.2023.2285868

**Published:** 2023-11-27

**Authors:** Yedong Yu, Caifeng Zhu, Yi Lin, Qian Qian, Xiaogang Shen, Wenli Zou, Minmin Wang, Jianguang Gong, Maosheng Chen, Lin Liu, Rizhen Yu, Quanquan Shen, Lina Shao, Bin Zhu

**Affiliations:** a Urology & Nephrology Center, Department of Nephrology, Zhejiang Provincial People’s Hospital, Affiliated People’s Hospital, Hangzhou Medical College, Hangzhou, Zhejiang, China; b Department of Nephrology, Hangzhou Traditional Chinese Medicine Hospital Affiliated to Zhejiang Chinese Medical University, Hangzhou, Zhejiang, China; c Department of Nephrology, Lin’an Hospital of Traditional Chinese Medicine, Hangzhou, Zhejiang, China

**Keywords:** Diabetic nephropathy, diabetic kidney disease, D-dimer, end-stage renal disease

## Abstract

**Background:**

This study aimed to investigate the relationship between plasma D-dimer levels, clinicopathological features, and clinical outcomes in patients with biopsy-proven diabetic nephropathy (DN).

**Methods:**

A total of 137 patients with biopsy-proven DN were enrolled in this two-center cohort study. Patients were stratified into tertiles based on plasma D-dimer levels. We investigated the relationship between plasma D-dimer levels and clinical outcomes, including a composite of death, a 40% decline in estimated glomerular filtration rate (e-GFR) from baseline, or end-stage renal disease (ESRD) (defined as e-GFR < 15 mL/min/1.73 m^2^ or need for renal replacement therapy including hemodialysis, peritoneal dialysis, or kidney transplantation), assessed using Cox regression models with adjustment for confounders.

**Results:**

At baseline, the mean age was 52.61 ± 11.63 years, and the mean e-GFR was 58.02 ± 28.77 mL/min/1.73 m^2^. During a median 26-month follow-up period, 65 (47% of patients) achieved clinical outcomes. Compared with the low plasma D-dimer level group, those with higher plasma D-dimer levels were more likely to have higher 24-h proteinuria (*p* = .002), lower e-GFR (*p* = .001), lower hemoglobin (*p* = .001), a higher glomerular lesion class (*p* = .03), and higher interstitial fibrosis and tubular atrophy (IFTA) scores (*p* = .002). After adjustment for demographic, DN-specific covariates, and treatments, it was observed that a higher tertile of plasma D-dimer was nonlinearly associated with an increased risk of the clinical outcomes (Hazard Ratio (HR) for tertile 2 vs. 1, 1.7; 95% Confidence Interval (CI), 0.80–3.75; HR for tertile 3 vs. 1, 2.2; 95% CI, 0.93–5.27; *p* for trend = .001) in the Cox proportional hazards models.

**Conclusion:**

In this study, DN patients with higher levels of plasma D-dimer had higher 24-h proteinuria, lower e-GFR, a higher glomerular lesion class, and higher IFTA scores. Furthermore, a high level of plasma D-dimer was nonlinearly associated with DN progression.

## Introduction

The prevalence of diabetes mellitus (DM) is increasing worldwide. According to the 2021 International Diabetes Federation (IDF) report, approximately 537 million people are diagnosed with DM, and this number is expected to reach 643 and 783 million by 2030 and 2045, respectively [1]. Also, the prevalence of DM was 9.5% in those without chronic kidney disease (CKD) and 35.6% in those with CKD between 2017 and March 2020 [2]. Diabetic nephropathy (DN) is a major microvascular complication in DM patients and a leading cause of end-stage renal disease (ESRD) [3]. Then, it is essential to identify risk factors associated with DN progression.

DN patients present with a hypercoagulability status. Hypercoagulability in DN is likely determined by multiple factors. For example, the renin-angiotensin-aldosterone system (RAAS) activates tissue factors (TF, factor III), leading to an increased incidence of thrombotic events. Moreover, hyperglycemia, dyslipidemia, and endothelial dysfunction are involved in the pro-thrombotic state of DN pathogenesis [4]. On vascular endothelial cell injury, the coagulation system and fibrinolytic activity are enhanced in patients with DN [5]. The tissue factor and downstream coagulation factors, such as active factor X (FXa), can activate protease-activated receptors (PARs), which subsequently exacerbate inflammation. Both PAR1 and PAR2 play a role in aggravating vascular inflammation in DN and exacerbating kidney injury by promoting cytokines and chemokines [6].

Plasma D-dimer is a soluble fibrin degradation product of the breakdown of thrombi. It serves as a valuable marker for the activation of coagulation and fibrinolysis [7]. Several studies have identified an association between increased plasma D-dimer levels and increased urinary albumin excretion (UAE) levels, as well as reduced e-GFR, in patients with DM [8–10]. However, the relationship between plasma D-dimer levels and clinical outcomes in patients with DN remains unknown. The present study was performed to determine the association between plasma D-dimer levels and disease progression in patients with biopsy-proven DN.

## Methods

### Study population and design

All patients with biopsy-proven DN at the initial renal biopsy between January 1, 2014, and August 31, 2022, were screened. DM was diagnosed according to the American Diabetes Association criteria [11]. The inclusion criterion was biopsy-proven DN with CKD stages 1–4. The exclusion criteria were as follows: coexistent systemic or nondiabetic renal disease; incomplete data; history of kidney transplantation; and presence of ESRD at the time of renal biopsy. The indications for renal biopsy in patients with DM included a short duration of diabetes, glomerular hematuria, sudden-onset overt proteinuria or increments in serum creatinine alone or in combination [12]. Hematuria is defined as a test result of five or more red blood cells per high-power field on urinary sediment. A common cutoff value for defining proteinuria is a protein-to-creatinine ratio greater than 0.3 mg/mg or 24-h urine protein excretion exceeding 150 mg. Patients were stratified into tertiles based on plasma D-dimer levels.

### Clinical and laboratory characteristics

The following data were collected at the time of renal biopsy: age, sex, body mass index (BMI), mean arterial pressure (MAP), diabetes duration, and laboratory tests, including glycosylated hemoglobin A1C (HbA1c), white blood cell (WBC), hemoglobin (Hb), platelet (PLT), prothrombin time (PT), activated partial thromboplastin time (APTT), international normalized ratio (INR), fibrinogen (FIB), albumin (ALB), uric acid, triglyceride, total cholesterol, low-density lipoprotein cholesterol (LDL-C), high-density lipoprotein cholesterol (HDL-C), serum creatinine, e-GFR, and 24-h proteinuria. The plasma D-dimer samplings were obtained on the morning of the renal biopsy day. Plasma D-dimer levels were measured using a latex particle-enhanced immunoturbidimetric assay with an automated coagulometer. The normal value of plasma D-dimer at our institution was ≤ 550 µg/l. Serum creatinine levels were collected to calculate the e-GFR, which was estimated using the creatinine-based Chronic Kidney Disease Epidemiology Collaboration equation [13,14]. All renal biopsies were performed with the patient’s consent.

### Histopathology

Renal biopsies were routinely performed for light microscopy and immunofluorescence to detect the renal pathological classification. Renal tissues were fixed in a 10% buffered formalin and embedded in paraffin. Two-micrometer-thick sections were stained with hematoxylin and eosin, trichrome, periodic acid-silver methenamine, and periodic acid Schiff to enable light microscopy analysis. Six-micrometer sections were stained using fluorescein-conjugated antibodies specific for human IgG, IgM, IgA, C3, C4, and C1q for immunofluorescence analysis. All sections were independently evaluated by two pathologists, blinded to the clinical data. In summary, glomerular lesions for DN were categorized following the criteria of the Research Committee of the Renal Pathological Society (RPS) [15]: Class I, featuring thickened glomerular basement membrane as observed by electron microscopy; Class II, displaying mild and severe mesangial expansion; Class III, indicating nodular sclerosis with less than 50% global glomerulosclerosis; and Class IV, representing more than 50% global glomerulosclerosis. Due to the unavailability of renal tissues examined by electron microscopy, we excluded class I from our analysis. Furthermore, the severity of IFTA was graded as follows: 0, no IFTA; 1, IFTA < 25%; 2, IFTA 25%–50%; 3, IFTA > 50%. In addition, global glomerulosclerosis and Kimmelstiel–Wilson (KW) lesions were assessed in this study.

### Outcomes

The clinical outcomes included a composite of death, a 40% decline in e-GFR from baseline, or ESRD (defined as e-GFR <15 mL/min/1.73 m^2^ or need for renal replacement therapy including hemodialysis, peritoneal dialysis, or kidney transplantation).

### Statistical methods

For continuous variables, values are expressed as mean ± standard deviation (SD) for normally distributed data and are compared using analysis of variance (ANOVA) among three groups. Non-normally distributed data are expressed as the median (interquartile range, 25%–75%) and are compared using the Kruskal–Wallis *H* test. Categorical variables are presented as ratios.

We conducted a Kaplan-Meier survival analysis to compare survival rates using the log-rank test. Furthermore, the association between plasma D-dimer levels and outcomes was evaluated using Cox proportional hazards models. The initial analysis was performed without any adjustments, followed by subsequent adjustments for several groups of baseline covariates that were chosen based on their clinical relevance and included in the model. Model 1 was adjusted for age and sex, the demographic characteristics of the participants. Model 2 included clinical risk factors related to DN progression: 24-h proteinuria, e-GFR, hemoglobin, cholesterol, and LDL-C, in addition to the variables from Model 1. Model 3 further included pathological parameters: glomerular lesion class, IFTA score, percentage of global glomerulosclerosis, and KW lesions, along with the variables from Model 2. Model 4 accounted for the covariates from Model 3 plus the treatment regimen, including the use of angiotensin-converting enzyme inhibitors (ACEIs) and angiotensin receptor blockers (ARBs). Based on the pathological findings, we stratified the glomerular lesion class and IFTA scores into categories II/III-IV and 1-2/3, respectively. Subgroup analyses were conducted within groups stratified by sex, age, proteinuria, e-GFR, BMI, MAP, hemoglobin, and pathological findings. Continuous variables of age, proteinuria, e-GFR, BMI, MAP, and hemoglobin were dichotomized using median values of 53 years, 2.5 g/24 h, 60 mL/min/1.73 m^2^, 24.5 kg/m^2^, 102 mmHg and 110 g/l, respectively, for subgroup analyses. All statistical analyses were performed using SPSS software (version 26.0) and the *R* programming language (version *R* 3.5.1). Differences were considered statistically significant at *p* < .05.

## Results

**Figure 1.**
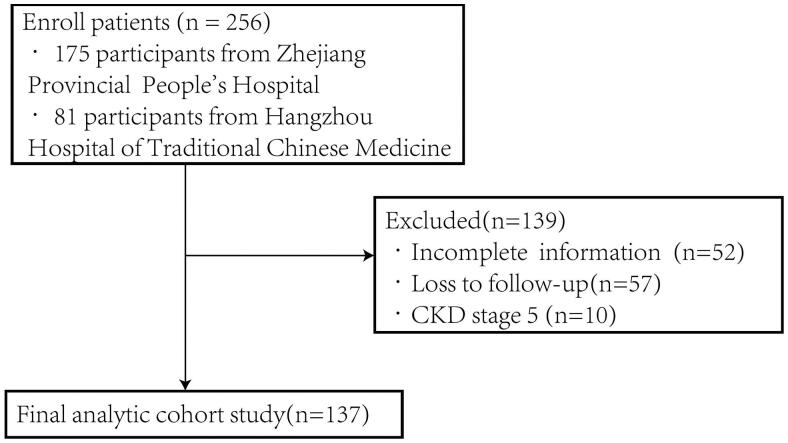
Flowchart for recruitment procedures.

We screened a total of 256 patients with biopsy-proven DN between January 1, 2014, and August 31, 2022, in the Department of Nephrology at Zhejiang Provincial People’s Hospital and Hangzhou Hospital of Traditional Chinese Medicine. Finally, 137 patients were included based on the inclusion and exclusion criteria (Figure 1).

### Baseline characteristics of the participants

At baseline (Table 1), the mean age of the participants was 52.61 ± 11.63 years, with males comprising 75% of the cohort. The mean e-GFR was 58.02 ± 28.77 mL/min/1.73 m^2^. The median duration of DM was 108 months (48, 156 months) and the median baseline plasma D-dimer level was 450 µg/L (245, 1065 µg/L). In terms of RPS classification, the percentages of Class II, III, and IV were 30.7%, 48.2%, and 21.2%, respectively. The IFTA scores of 1, 2, and 3 accounted for 16.8%, 53.3%, and 29.9%, respectively. KW lesions were found in 54.7% of the participants. Immunofluorescence revealed immune deposits in 85 patients (62.0%), with IgM deposits being the most common pattern (51.8%).

**Table 1.** Baseline characteristics of 137 diabetic nephropathy patients according to plasma D-dimer tertiles.

		Plasma D-dimer, ug/L			
Characteristics	All participants	T1:<340	T2:340–780	T3:>780	*p* value
Subjects	137	46	45	46	
Age(years)	52.6 ± 11.6	51.1 ± 11.6	52.0 ± 10.6	54.8 ± 12.3	.439
Sex/male (*n*, %)	103(75.2%)	34(73.9%)	31(68.9%)	38(82.6%)	.311
BMI (kg/m^2^)	24.7 ± 3.0	24.8 ± 3.0	24.9 ± 2.9	24.5 ± 3.2	.763
Diabetes duration (months), median (IQR)	108(48,156)	120 (60,153)	108(60,145)	72(25,156)	.276
Smoking (*n*, %)	54(39.4%)	18(39.1%)	16(35.6%)	20(43.5%)	.742
MAP (mmHg)	103.4 ± 14.1	100.4 ± 13.9	103.5 ± 14.5	106.1 ± 13.2	.084
HbA1c (%)	7.4 ± 1.8	7.5 ± 1.5	7.3 ± 2.1	7.4 ± 1.8	.35
24-h proteinuria(g), median (IQR)	2.58(1.01,4.67)	1.35(0.65, 3.44)	2.58(1.23,4.46)	3.67(1.94, 6.03)	.002
WBC(×10^9^/L)	6.8 ± 2.0	7.0 ± 2.3	6.9 ± 2.1	6.5 ± 1.6	.633
Hb (g/d)	111.5 ± 21.7	121.9 ± 21.7	108.9 ± 21.3	103.8 ± 17.9	.001
PLT(×10^9^/L)	210.3 ± 65.4	216.3 ± 58.9	202.4 ± 64.1	211.9 ± 71.7	.64
PT(s)	11.2 ± 1.0	11.0 ± 0.7	11.2 ± 1.0	11.5 ± 1.1	.038
INR	1.00 ± 0.10	0.98 ± 0.07	1.00 ± 0.09	1.04 ± 0.12	.012
APTT(s)	27.6 ± 5.4	27.5 ± 3.4	27.2 ± 3.1	28.0 ± 8.2	.836
FIB(g/L)	3.9 ± 1.2	3.4 ± 0.7	4.0 ± 1.1	4.3 ± 1.4	.001
ALB(g/L)	33.0 ± 6.1	36.4 ± 5.0	33.4 ± 5.8	29.3 ± 5.1	.001
Uric acid (umol/L)	393.2 ± 93.2	399.5 ± 77.7	381.8 ± 99.3	398.2 ± 99.1	.612
Triglycerides(mmol/L)	2.1 ± 1.7	2.0 ± 1.5	2.3 ± 2.1	2.0 ± 1.1	.84
Cholesterol(mmol/L)	5.0 ± 1.6	4.7 ± 1.3	4.8 ± 1.3	5.6 ± 1.8	.019
HDL-C(mmol/L)	1.1 ± 0.4	1.1 ± 0.3	1.1 ± 0.4	1.1 ± 0.4	.511
LDL-C(mmol/L)	2.9 ± 1.2	2.6 ± 1.0	2.8 ± 1.1	3.3 ± 1.3	.006
eGFR (mL/min/1.73 m^2^)	58.0 ± 28.8	70.6 ± 27.2	55.9 ± 27.1	47.5 ± 27.1	.001
Use of ACEI/ARB (*n*, %)	80(58.4%)	34(73.9%)	25(55.6%)	21(45.7%)	.021
Pathological findings					
Glomerular lesion class, *n* (%)					.03
II	42(30.7%)	20(43.5%)	10(22.2%)	12(26.1%)	
III	66(48.2%)	20(43.5%)	21(46.7%)	25(54.3%)	
IV	29(21.2%)	6(13.0%)	14(31.1%)	9(19.6%)	
IFTA score, *n* (%)					.002
1	23(16.8%)	12(26.1%)	3(6.7%)	8(17.4%)	
2	73(53.3%)	29(63.0%)	26(57.8%)	18(39.1%)	
3	41(29.9%)	5(10.9%)	16(35.6%)	20(43.5%)	
Global glomerulosclerosis, %	30.9 ± 20.8	27.3 ± 18.1	36.2 ± 21.7	29.3 ± 21.4	.139
Kimmelstiel–Wilson lesion, present, *n* (%)	75(54.7%)	21(45.7%)	26(57.8%)	28(60.9%)	.304
Immune deposits, *n* (%)	85(62.0%)	28(60.9%)	31(68.9%)	26(56.5%)	.471
IgM, *n* (%)	71(51.8%)	22(47.8%)	27(60%)	22(47.8%)	.411
IgA, *n* (%)	30(21.9%)	8(17.4%)	13(28.9%)	8(17.4%)	.375
IgG, *n* (%)	35(25.5%)	14(30.5%)	12(26.7%)	9(19.6%)	.481
C3, *n* (%)	34(24.8%)	13(28.3%)	11(24.4%)	10(22.2%)	.769
C4, *n* (%)	15(10.9%)	6(13%)	4(8.9%)	5(10.9%)	.819
C1q, *n* (%)	19(13.9%)	9(19.6%)	6(13.3%)	4(8.7%)	.321

Continuous variables are expressed as mean ± SD or as median (interquartile range). Categorical variables are expressed as frequency (percent).

MAP: mean artery pressure; HbA1C: glycosylated hemoglobin A1C; eGFR: estimated glomerular filtration rate; WBC: white blood cell; Hb: hemoglobin; PLT: platelet; PT: Prothrombin time; INR: international normalized ratio; APTT: activated partial thromboplastin time; FIB: Fibrinogen; ALB: albumin; HDL-C: High-Density Lipoprotein Cholesterol; LDL-C: Low-Density Lipoprotein Cholesterol; ACEI: angiotensin-converting enzyme inhibitor; ARB: angiotensin receptor blocker; IFTA: interstitial fibrosis and tubular atrophy; T: tertile.

The clinicopathological characteristics, stratified into tertiles based on plasma D-dimer levels are summarized in Table 1. There were no significant differences in age, sex, diabetes history, MAP, smoking history, HbA1c, WBC, PLT, uric acid, triglyceride, HDL-C, global glomerulosclerosis, KW lesion, or immune deposits among the three baseline plasma D-dimer tertile groups. However, patients with higher plasma D-dimer levels were more likely to have higher 24-h proteinuria (*p* = .001), higher total cholesterol (*p* = .019), higher LDL-C (*p* = .006), lower hemoglobin (*p* = .002), and lower e-GFR (*p* = .001). Additionally, there was a lower percentage of patients using RAAS inhibitors (ACEIs/ARBs) in the higher plasma D-dimer tertile groups (*p* = .021). Patients in the lowest-level plasma D-dimer tertile group presented with less pathological injury (56% with class III-IV glomerular lesions and 74% with IFTA scores of 2–3) as compared with the other two high-level groups (78% with class III-IV glomerular lesions and 93% with IFTA scores of 2–3 in the second tertile group, 74% with class III-IV glomerular lesions, and 83% with IFTA scores of 2–3 in the third tertile group).

After adjusting for age and sex, plasma D-dimer levels were positively associated with the IFTA score (*r* = 0.261, *p* = .002) and negatively correlated with e-GFR (r = −0.316, *p* = .001). Moreover, after adjusting for age, sex, and e-GFR, plasma D-dimer levels were positively correlated with 24-h proteinuria (*r* = 0.224, *p* = .009), cholesterol (*r* = 0.281, *p* = .009), and LDL-C (*r* = 0.291, *p* < .001), while they were negatively correlated with hemoglobin (r = −0.333, *p* < .001) and serum albumin (*r* = −0.485, *p* < .001) (Table 2).

**Table 2.** Correlations between the plasma D-dimer level and clinical–histopathological findings.

	Variables	Correlation coefficient (r)	*p* value
Plasma D-dimer	IFTA	0.261	.002^a^
	e-GFR (mL/min/1.73 m^2^)	−0.316	.001^b^
	24-h proteinuria(g)	0.224	.009^c^
	Hemoglobin(g/L)	−0.333	.001^c^
	Cholesterol(mmol/L)	0.281	.001^c^
	LDL-C(mmol/L)	0.291	.001^c^
	Serum albumin(g/L)	−0.485	.001^c^

IFTA: interstitial fibrosis and tubular atrophy.

^a^ Spearman’s correlation analysis. A two-tailed *p* < .05 was considered statistically significant.

^b^ Partial correlation analysis for adjusting the baseline age and sex.

^c^ Partial correlation analysis for adjusting the baseline age, sex and e-GFR.

#### Plasma D-dimer levels and outcomes

**Figure 2.**
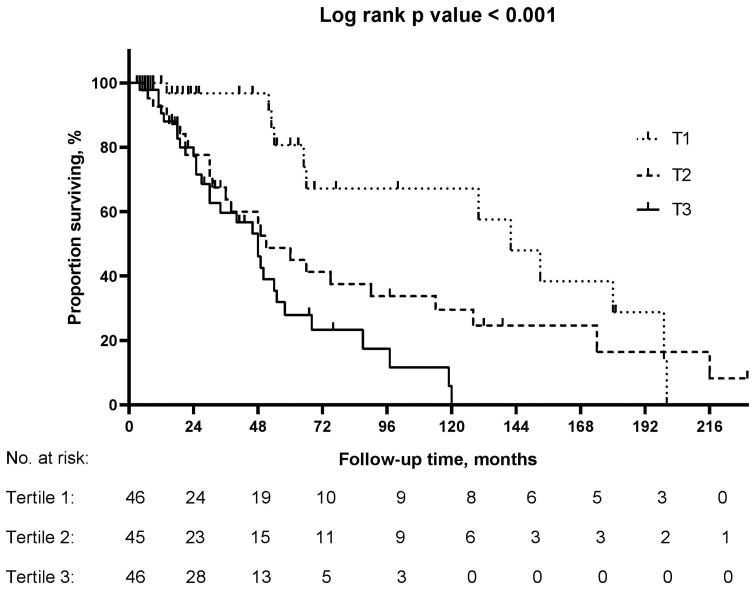
A Kaplan–Meier survival curve demonstrated that the cumulative survival rate for the clinical outcomes was lower in patients with high tertile of plasma D-dimer compared to those in the low tertile group. (*p* < .001). T: tertile.

In total, 137 patients (70.6%) were available for follow-up. At the end of the follow-up period, 65 cases (47%) of participants (6 deaths, 41 cases with dialysis, 8 cases with e-GFR < 15 mL/min/1.73 m^2^, and 10 cases with a reduction in e-GFR by 40% from baseline) reached the clinical outcomes during a median follow-up of 26 months. The cumulative incidence proportions of the clinical outcomes were 26.1%, 53.3%, and 63.0% in the first, second, and third plasma D-dimer tertile groups, respectively. Kaplan–Meier analyses showed that patients with higher plasma D-dimer levels had a significantly higher risk of developing clinical outcomes (log-rank *p* < .001; Figure 2).

**Figure 3.**
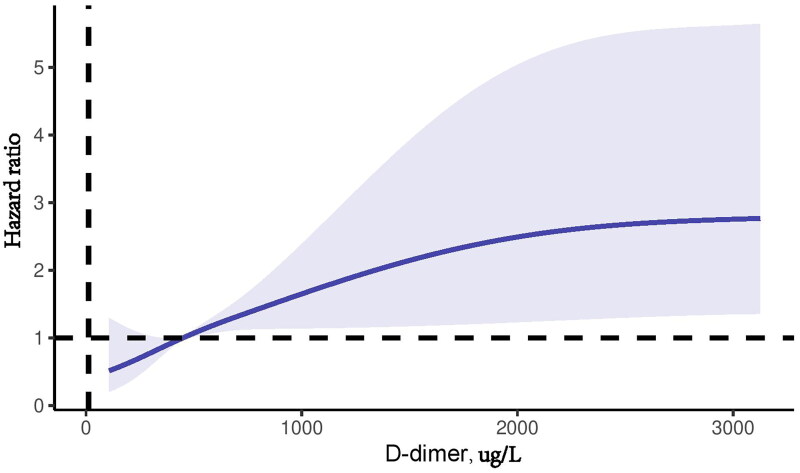
Restricted cubic spline analysis shows a nonlinear relationship between the plasma D-dimer levels and the risk of developing clinical outcomes in the DN participants using the values of the 5th, 35th, 65th, and 95th percentiles of plasma D-dimer levels as the knots.

As shown in Figure 3, restricted cubic spline analysis demonstrated a nonlinear relationship between plasma D-dimer levels and clinical outcomes. The risk of developing clinical outcomes increased gradually with the rise in plasma D-dimer levels up to 456 ug/L; Subsequently, the risk increased more rapidly. We then examined the relationship between plasma D-dimer levels and outcomes using Cox proportional hazards models (Table 3), with the first tertile group as the reference. In the unadjusted analysis, high plasma D-dimer levels appeared to be associated with an increased risk of developing clinical outcomes (model 1, *p* for trend < .001; HR for tertile 2 vs. 1, 2.1; 95% CI, 1.03–4.18; HR for tertile 3 vs. 1, 4.0; 95% CI, 1.95–8.13). In the fully adjusted model, the association between a high plasma D-dimer level and a high cumulative incidence of the clinical outcomes remained (model 4, P for trend = 0.001; HR for tertile 2 vs. 1, 1.7; 95% CI, 0.80–3.75; HR for tertile 3 vs. 1, 2.2; 95% CI, 0.93–5.27).

**Table 3.** Hazard ratios and 95% confidence intervals of clinical outcomes according to tertile of plasma D-dimer among DN patients.

		Tertile of plasma D-dimer		*p* for trend
	T1:<340 µg/L	T2:340–780	T3:>780 µg/L	
N	46	45	46	
Median of plasma D-dimer, ug/L	193.5	450.0	1480.0	
Outcomes, *n*	12	24	29	
Crude	1.0	2.1(1.03,4.18)	4.0(1.95,8.13)	<.001
Model 1	1.0	2.1(1.04,4.25)	3.9(1.88,8.06)	<.001
Model 2	1.0	2.0(0.91,4.16)	2.9(1.30,6.58)	<.001
Model 3	1.0	1.8(0.86,4.00)	2.5(1.05,5.82)	.001
Model 4	1.0	1.7(0.80,3.75)	2.2(0.93,5.27)	.001

Clinical outcomes including a composite of death, 40% decline in eGFR or ESRD (defined as eGFR <15 mL/min/1.73 m^2^ or need for kidney replacement therapy including hemodialysis, peritoneal dialysis, or kidney transplantation).

【Model 1, adjusted for age, sex; 】.

【model 2, adjusted for age, sex, hemoglobin, 24-h proteinuria, eGFR, TC and LDL-C;】.

【model 3, adjusted for age, sex, hemoglobin, 24-h proteinuria, eGFR, TC, LDL-C,, Glomerular lesion class, IFTA score, Percentage of global glomerulosclerosis, Kimmelstiel–Wilson lesion (absent/present);】.

【model 4, adjusted for age, sex, hemoglobin, 24-h proteinuria, eGFR, TC, LDL-C,, Glomerular lesion class, IFTA score, Percentage of global glomerulosclerosis, Kimmelstiel–Wilson lesion (absent/present) and treatments ACEIs/ARBs. 】.

### Subgroup analyses

Subgroup analyses were stratified by age, sex, BMI, smoking status, hemoglobin levels, MAP, e-GFR, proteinuria, cholesterol, LDL-C, glomerular lesion classification, IFTA score, Kimmelstiel-Wilson lesion, and ACEI/ARB usage (Table 4). Consistent with the primary analyses, higher plasma D-dimer levels were significantly associated with an increased risk of clinical outcomes in most subgroups. However, no significant interactions were observed between risk factors and plasma D-dimer levels. Additionally, there was no significant asso­ciation between plasma D-dimer levels and the clinical outcomes in the patients with hemoglobin ≥ 110 g/L, proteinuria < 2.5 g/24 h, e-GFR ≥ 60 mL/min/1.73 m^2^, IFTA score of 3, and the use of ACEI/ARB.

**Table 4.** HRs and 95% CIs of clinical outcomes according to tertiles of plasma D-dimer: subgroup analyses.

	Number	Tertile of plasma D-dimer(range)				
		T1	T2	T3	*p* trend	*P* interaction
Total participants	137	1.0	2.1(1.03,4.18)	4.0(1.95,8.13)	<.001	
**Age, years, median**						.292
≥53	70	1.0	2.0(0.39,10.47)	5.5(1.20,25.36)	.018	
<53	67	1.0	2.4(1.10,5.26)	4.0(1.67,9.53)	.004	
**Gender**						.717
Female	34	1.0	1.1(0.76,5.53)	5.4(1.50,19.44)	.024	
Male	103	1.0	3.4(1.33,8.75)	4.0(1.56,10.12)	.003	
**BMI, m^2^/kg, median**						.288
≥24.5	70	1.0	3.0(0.97,9.39)	5.0(1.57,15.93)	.010	
<24.5	67	1.0	1.5(0.61,3.81)	3.4(1.36,8.63)	.019	
**Smoking**						.542
yes	54	1.0	3.3(0.87,12.44)	4.4(1.25,15.23)	.029	
no	83	1.0	1.9(0.79,4.48)	4.1(1.62,10.4)	.008	
**MAP, mmHg, median**						.631
≥102	68	1.0	2.7(0.97,7.65)	4.9(1.80,13.14)	.003	
<102	69	1.0	1.9(0.7,4.96)	3.1(1.08,9.08)	.097	
**Hemoglobin, g/L, median**						.255
≥110	69	1.0	1.0(0.32,3.45)	2.4(0.73,8.16)	.3	
<110	68	1.0	2.9 (1.13,7.31)	4.6 (1.76,11.83)	.002	
^a^ **Cholesterol, mmol/L**						.386
≥5.7	41	1.0	1.8(0.53,6.50)	5.9(1.63,21.19)	.007	
<5.7	96	1.0	2.5(1.0,5.98)	4.1(1.65,10.26)	.006	
^a^ **LDL-C, mmol/L**						.542
≥3.1	48	1.0	2.0(0.52,7.68)	5.5(1.45,21.19)	.01	
<3.1	89	1.0	2.3(0.98,5.53)	3.9(1.59,9.71)	.008	
**Proteinuria, mg/24 h, median**						.623
≥2500	69	1.0	2.3(0.93,5.61)	3.8(1.55,9.39)	.007	
<2500	68	1.0	1.6(0.53,5.11)	4.0(1.20,13.27)	.066	
^b^ **eGFR, mL/min**						.583
≥60	57	1.0	3.2(0.99,10.39)	4.8(1.09,21.63)	.051	
<60	80	1.0	1.9(0.73,5.11)	3.3(1.31,8.15)	.018	
**RPS, glomerular classification**						.083
Class II	42	1.0	1.9(1.09,17.92)	13.6(9.09,30.33)	.000	
Class III/IV	95	1.0	1.4(0.66,3.10)	2.6(1.22,5.59)	.027	
**IFTA**						.844
1/2	96	1.0	1.6(0.7,3.79)	3.4(1.35,8.54)	.031	
3	41	1.0	3.9(0.82,18.88)	3.7(0.83,16.06)	.105	
**Kimmelstiel–Wilson lesion**						.133
Present	75	1.0	1.3(0.59,2.83)	2.6(1.20,5.74)	.031	
Absent	62	1.0	1.5(0.85,4.47)	2.2(1.11,5.34)	.012	
**ACEI/ARB**						.229
Yes	80	1.0	1.9(0.84,4.44)	2.6(0.97,7.16)	.116	
No	57	1.0	2.7 (0.58,12.61)	5.0 (1.12,22.17)	.029	

^a^ The cutpoint of Cholesterol and LDL-C were the value of *clinical* *cut*‐*off*.

^b^ The cutpoint of 60 mL/min between CKD stages II and III that was close to the median (58.0).

## Discussion

In this two-center retrospective cohort study, we revealed that DN with higher plasma D-dimer levels had poorer renal function and more severe glomerular lesions. Furthermore, our findings revealed a nonlinear association between plasma D-dimer level and DN progression. Notably, the risk of developing clinical outcomes increased rapidly when the plasma D-dimer level exceeded 456 ug/L.

Vascular endothelial cells (ECs) are particularly susceptible to damage from chronic hyperglycemia-induced inflammation. Subendothelial collagen is fully exposed, which results in platelet aggregation and activation of the coagulation system. Plasma D-dimer was initially considered a specific degradation product of fibrin, which reflects hypercoagulation and fiber activity. The hypercoagulable state is characterized by increased levels of various pro-thrombotic markers, among which plasma D-dimer is the gold standard. Currently, hypercoagulability plays a major role in DN progression [16,17]. And endothelial dysfunction plays a critical role in hypercoagulability in DN. In contrast, hypercoagulability exacerbates DN progression. Impaired endothelial nitric oxide synthase (eNOS) production or reduced eNOS activity represents a hallmark of endothelial dysfunction in DN. Impaired eNOS expression is likely to be associated with an increase in TF-dependent coagulation [18]. The coagulation cascade involving factor Xa (FXa), located downstream of TF/VIIa, can activate both PAR1 and PAR2. These receptors, in turn, promote the expression of inflammatory mediators such as monocyte chemotactic protein 1 (MCP1), plasminogen activator inhibitor-1 (PAI-1), and pro-fibrotic molecules in endothelial, mesangial, and renal tubular cells [19–21]. Therefore, we speculated that higher plasma D-dimer levels may indicate more severe microangiopathy-induced damage to endothelial cells. The abnormal activation of the coagulation and fibrinolytic systems, along with the release of various vasoconstricting substances, such as endothelin, triggers a cascade of subsequent inflammatory cytokine reactions that accelerate the DN progression.

The association between plasma D-dimer levels and renal dysfunction in DN may stem from an increase in plasma D-dimer synthesis rather than a reduction in urinary D-dimer excretion. It is noteworthy that DN patients with proteinuria have higher levels of urinary D-dimer than healthy individuals [22]. Natural anticoagulants such as antithrombin, protein C, and protein S can be lost *via* proteinuria, further exacerbating the hypercoagulable state and promoting plasma D-dimer production [23].

As a pathological disorder, CKD is characterized by chronic inflammation and hypercoagulability. Inflammation, by altering adhesion molecules on the endothelium and platelets, affects the vascular wall and activates leukocytes that perpetuate damage, thus leading to heightened inflammation and hypercoagulability [24,25]. Similarly, DN shares common features with CKD, exhibiting chronic inflammation and hypercoagulability [26]. Despite the widespread use of RAAS inhibitors and hypoglycemic drugs, most patients with DN eventually progress to ESRD. Recent reports have highlighted that elevated levels of FXa and PAR2 exacerbate DN progression, making both FXa and PAR2 promising therapeutic targets for the management of DN [19].

No significant modifications by any subgroup were observed in the effects of plasma D-dimer on DN progression. In the subgroup with MAP < 102 mmHg, plasma D-dimer levels showed no prominent association with outcomes. This observation may reflect the influence of other detrimental factors such as sex (HR 0.201, 95% CI 0.048–0.838; P for trend = 0.028) and IFTA (HR 5.356, 95% CI 1.221–23.499; P for trend = 0.026), which outweighed the potentially harmful effects of plasma D-dimer (HR for tertile 3 vs. 1, 0.948; 95% CI 0.232-3.870; HR for tertile 2 vs. 1, 1.245; 95% CI 0.358–4.33; P for trend = 0.903) on kidney function in patients with lower MAP. In the subgroup with hemoglobin ≥ 110 g/L, the association between plasma D-dimer levels and the outcomes was not statistically significant. This observation can be attributed to the fact that in patients with anemia, the detrimental effects of plasma D-dimer may be more pathogenic and easier to discern compared to those without anemia. Meanwhile, other factors that promote plasma D-dimer elevation and contribute to poor outcomes might have a more significant impact. Several studies have indeed demonstrated that chronic anemia increases patients’ susceptibility to hypercoagulability [27,28]. Interstitial fibrosis and tubular atrophy have been demonstrated to be an essential independent risk factor for DN progression [29]. In the present study, the factor may outweigh the deleterious effects of plasma D-dimer in the subgroup with a high IFTA score (IFTA 3). Additionally, it is worth noting that some studies have shown that cigarette smoking is not a significant risk factor for adverse outcomes [30]. In our study, the percentage of smokers in the first tertile was not significantly lower than those in other tertiles. These findings provide further insight into the potential association between plasma D-dimer levels and clinical outcomes in patients with DN. While it is true that some HR with 95% CI range from < 1 to > 1 and have individual *p*-values that appear insignificant, our primary focus is on the p-values for trend. In Tables 3 and 4, we evaluate the *p*-values for trend to assess the overall significance of the associations, considering the direction and consistency of the effects across groups.

The strengths of this study were the large sample size, two study centers, a long follow-up period, and inclusion of DN-specific risk factors for the multivariable adjustment analyses. However, there were some limitations to this study. This was a retrospective cohort study, and a causal relationship between plasma D-dimer levels and renal prognosis could not be established. Due to the unavailability of electron microscopy, the diagnosis of class I DN was not feasible, resulting in an underestimation of DN. Large-scale prospective biopsy studies are needed to determine whether a reduction in plasma D-dimer levels has a beneficial effect on DN pathology and long-term outcomes.

In conclusion, our study has illuminated that DN patients with higher levels of plasma D-dimer had higher 24-h proteinuria, lower e-GFR, a higher glomerular lesion class, higher IFTA scores, and demonstrated a nonlinear association with DN progression. It was noticeable that the association between higher plasma D-dimer levels and DN progression was less significant in patients with lower MAP, higher hemoglobin, and more severe IFTA, which may be due to some more confounding factors in association with DN progression in these patients. Future prospective studies are warranted to confirm these findings and investigate the underlying mechanisms.
